# Charting the Course of the Nursing Professional Identity: A Qualitative Descriptive Study on the Identity of Nurses Working in Care for Older Adults

**DOI:** 10.1111/jan.16506

**Published:** 2024-10-02

**Authors:** Ester Ellen Trees Bolt, Shi Yin Chee, Margreet van der Cingel

**Affiliations:** ^1^ Leeds University Business School, University of Leeds Leeds UK; ^2^ Faculty of Social Sciences and Leisure Management Taylor's University Subang Jaya Selangor Malaysia; ^3^ Active Ageing Impact Lab Taylor's University Subang Jaya Selangor Malaysia; ^4^ NHL Stenden University of Applied Sciences Leeuwarden the Netherlands; ^5^ Medical Centre Leeuwarden Leeuwarden the Netherlands

**Keywords:** healthcare systems, inductive content analysis, nurse identity, nurse turnover, older adults, professional identity

## Abstract

**Aims:**

To explore and describe the meaning of nurses working in care for older adults give to the nursing professional identity.

**Design:**

A qualitative approach was taken.

**Methods:**

Semi‐structured interviews were conducted with 50 bachelor and vocational‐educated nurses working in care for older adults. Interviews were conducted between December 2019 and May 2020. Data were analysed and interpreted through inductive content analysis.

**Results:**

Five themes embody the meaning of the nursing professional identity of nurses who work in care for older adults. The five themes are: born to care: a lifelong motivation to nursing; nursing through the noise: dedication in a demanding profession; the silent backbone: caught in the crossfire of interdisciplinary teams; learning under pressure: the demand for expanded nursing expertise and against the current: the barriers to advocacy in nursing.

**Conclusion:**

The professional nursing identity of nurses working in care for older adults is multi‐faceted. A personal dedication to patient care, where patients ‘human’ aspect is heavily valued, commits nurses to their profession and underscores their dedication to upholding the quality standard in nursing practice.

**Implications for the Profession:**

The older adults' nursing identity highlights that nursing deserves acknowledgement as a professional occupation. Nurses should speak to the public about their professional roles to improve the public view of older adult nursing.

**Impact:**

A clear understanding of the older adult nursing professional identity clarifies specific roles, experiences and expectations. This can help attract and retain nurses whose views of older adult nursing align with the nursing professional identity. This could help resolve nurse turnover and reduce shortages in older adult care.

**Reporting Method:**

We adhered to Consolidated Criteria For Reporting Qualitative Research guidelines.

**Patient or Public Contribution:**

No patient or public contribution.

**Summary**

- Why is this research needed?
  ○ Population ageing fuels care for older adults as people age without necessarily experiencing equivalent health improvements.
  ○ Nurses working in older adult care lead in addressing this surge by providing care to older adults amidst high nurse turnover and shortages.
  ○ Comprehending the nursing professional identity is vital to attracting and retaining nurses to older adult care and helps elevate the perception of nurses working in care for older adults as a respected profession.
- What are the key findings?
  ○ Participants described an identity that resonated with the person‐centred care philosophy but also shared experiences unique to caring for older adults, particularly in relation to the degree of power they enjoy in the care relationship.
  ○ Nurses in older adult care perform multifaceted roles within interdisciplinary teams but encounter recognition challenges from healthcare hierarchy and societal gender norms.
  ○ Participants emphasised advocacy and ongoing skill development to meet future care needs.
- How should the findings be used to influence policy/practice/research/education?
  ○ Healthcare organisations and nurses must enhance public perceptions of nursing in care for older adults.
  ○ The professional nursing identity presented in this study aids in recruiting individuals whose perspectives of nursing align, curbing unrealistic expectations.
  ○ Given the significant role nurses fulfil in interdisciplinary healthcare, policy development is needed that clearly defines their roles and expectations, preventing conflicts arising from multi‐level demands.

## 1 Introduction

Nurses are instrumental in preserving and promoting people's well‐being. Despite significant efforts by governments and healthcare organisations to stabilise the nursing workforce, the International Council of Nurses (2023) reports high turnover rates and shortages within the nursing occupation. In tandem with nurse shortages, demographic shifts such as ageing populations and increased care complexity present further challenges, particularly for geriatric and gerontological care in the Netherlands, where a shortage of over 67,000 nurses is predicted for nursing homes and extramural care by 2031 (ABF Research 2022). While improved living conditions have led to increased lifespans, they do not guarantee enhanced health, reinforcing the need for specialised care for older adults (Chee, Dasgupta, and Ari Nagavan 2023).

Older adult care nurses are positioned at the centre of these challenges as they provide day‐to‐day care to older adults. In the Netherlands, a shortage of 24,000 nurses in geriatric and gerontological care was already evident in 2022 (Dutch Ministry of Health 2023). The challenge is compounded by population ageing, with 20.2% of the Dutch population being over 65 years old in 2023 and a notable growth in adults over 80 (Statistics Netherlands 2023). Preparing to mitigate these challenges hinges on attracting and retaining nurses in older adult care settings, underscoring the importance of understanding the nursing professional identity of nurses working in care for older adults. A professional identity is a ‘construct that combines one's values and belief system with societal and professional elements’ (van der Cingel and Brouwer 2021, 4). However, the professional identity of nurses caring for older adults remains insufficiently explored.

Defining the professional identity of nurses working in care for older adults is important for three reasons. First, it facilitates nurses' comprehension of their job roles, mitigating unrealistic expectations and strengthening their identification with their professional identity. Second, a stronger identification of nurses with their nursing professional identity can benefit the older adult care sector as professional identity relates to meaningful organisational outcomes. Third, a better understanding of the professional identity of nurses working in care for older adults could enhance the public's perception of older adult nursing, elevating it to a profession deserving of greater respect and professionalism.

## 2 Background

The nursing occupation has long captivated the interest of researchers, with recent nursing studies investigating issues such as turnover (Bolt et al. 2024; Pahlevan Sharif et al. 2021), leadership (van der Cingel, Andela, and Barf 2023; Kitson 2023) and well‐being (Xiao, Cooke, and Chen 2022). Understanding the fundamental beliefs of employees is key to addressing organisational and work‐related issues. Several stereotypes continue to burden the nursing occupation. Historically, the public has perceived nursing as an occupation focused on bedside care and kindness (Cohen 2007). Nursing is also seen as a female, subservient and practical occupation (van der Cingel and Brouwer 2021), viewed as a supplementary occupation to medicine, encapsulated by the perception that ‘nurses care and doctors cure’ (Thompson and McNamara 2022, 843). These stereotypes hinder nurses and recruits from fully understanding their roles, which can reduce their commitment (Ten Hoeve, Brouwer, and Kunnen 2020) and retention (Bolt, Winterton, and Cafferkey 2022). van der Cingel and Brouwer (2021) argue that this perception is overly simplistic and does not fully represent nurses' true professional identity.

The ongoing debate has reignited researchers' interest in developing a nursing professional identity, a view advocated for by Florence Nightingale decades ago (Nightingale 1969). Care philosophies on the fundamentals of care (Kitson 2018) and person‐centred care (Morgan and Yoder 2012) describe nursing roles and relationships with patients and the model of Mayer et al. (2020) presents the Person‐centred Practice Framework specific to long‐term care. While developed as a relational philosophy of care, the model refers to aspects of professional identity that relate to nursing, such as being committed to the job and having a sympathetic presence. This emphasises how ‘the person of the nurse’ matters and underscores how care is defined by mutuality within the care relationship. The person‐centred care philosophy prioritises focusing on individual needs, values and preferences, emphasising the patient's active participation in their care to ensure it aligns with their needs and wants (Byrne, Baldwin, and Harvey 2020). However, while personal involvement can aid recovery, not all patients can actively participate or have recovery opportunities, especially in older adult care, where geriatric conditions limit involvement and recovery prospects.

For this study, the professional identity of nurses is defined as ‘a sense of oneself, and in relationship with others, that is influenced by characteristics, norms, and values of the nursing discipline, resulting in an individual thinking, acting and feeling like a nurse’ (Goodolf and Godfrey 2021, 495). This definition guided the researchers' exploration and the meanings attributed to professional identity in this study. To clarify, professional identity should be distinguished from care philosophies such as the fundamentals of care and person‐centred care, which focus primarily on care delivery and patient interaction. Professional identity encompasses a broader view, including nurses' self‐concept and role perception.

Researchers have explored the meanings attributed to nurses' professional identity from diverse perspectives (Pursio, Kankkunen, and Kvist 2023; Wu, Palmer, and Sha 2020). Maturing research indicates that nurses' professional identity is best described as intersecting personal and interpersonal aspects, influenced by gendered sociological–historical views that often portray nursing as a female profession (Öhlén and Segesten 1998). Stanley (2008) analysed nurse representation in 280 Western films, observing that many nurses were depicted as submissive workers with sexual and romantic features. Godsey, Houghton, and Hayes (2020) studied nurse identity through a marketing lens, identifying several issues in the public perception of nursing, including portraying the occupation as female‐dominated in the media. While these interpretations offer important insights, studies directly capturing nurses' perceptions shed a different light on their professional identity, depicting nurses as highly skilled and knowledgeable professionals.

Brzozowski, King, and Steege (2023) explored nurse identity in United States primary care settings, finding that primary care nurses described their professional identity as detectives, navigators and implementers. Nurses saw themselves as detectives when assessing patient needs, as navigators when helping patients move through the healthcare system to access necessary care and as implementers when providing medical care to patients. Thompson and McNamara (2022) explored the professional identity of Irish Advanced Nurse Practitioners (ANP) from the perspectives of various medical and healthcare professionals. Their study found that ANPs were perceived as valuable, knowledgeable and autonomous nurses, but they also faced occasional judgement for crossing hierarchical boundaries. Dahl and Clancy (2015) explored nurse identities by interviewing public health nurses, who considered their professional identity intricate and multifaceted, requiring comprehensive knowledge across various domains and a personalised focus on patient well‐being. Additionally, public health nurses found themselves absorbed in addressing the needs of individuals with specific conditions. The nursing profession was described as demanding; patients often have high expectations, expecting nurses to provide answers to all their inquiries.

In short, previous research found different aspects that delineate different identities for nursing in various domains. These various nursing domains require different nursing practices, which postulates the possibility of different nurse identities for nurses in these domains. These aspects underscore the significance of this study's focus on nurses working in care for older adults and their perceptions, meanings and experiences of their professional identity. The complexity and conflicting views coupled with nurse turnover and retention issues and the expected increased demand in older adult care present a timely opportunity to assess older adult care nurses' professional identity.

## 3 The Study

### 3.1 Aims

This study aims to understand and describe the nursing professional identity by exploring the meanings nurses working in care for older adults assign to their professional identity. The central research question was: ‘How do nurses working in care for older adults describe their nursing professional identity?’

### 3.2 Study Design

Owing to the study's exploratory scope and emphasis on participants' subjective experiences, a qualitative descriptive design (Graneheim and Lundman 2004) was chosen, using an inductive content analysis of transcribed interviews. This method, known for offering in‐depth, nuanced insights into individuals' viewpoints, perfectly aligns with the study's objective of revealing nurses' perceptions of their professional identities. This study followed the Consolidated Criteria for Reporting Qualitative Studies (COREQ), a 32‐item checklist designed to enhance transparency and rigour in reporting qualitative findings (Tong, Sainsbury, and Craig 2007), available in Supporting Information S1.

### 3.3 Participants and Data Collection

This study focused on understanding the various meanings older adult care nurses assign to their professional identity, so participants were purposefully selected. Eligibility criteria required participants to possess at least 1 year of occupational experience and to be actively working as older adult care nurses. The research team collaborated with the Human Resource (HR) departments of five well‐established older adult care organisations based in the Netherlands to facilitate participant recruitment. These organisations had a long history of providing care for older adults in intramural and extramural settings. The organisations offered various types of gerontological care, including palliative care, home care, dementia care, rehabilitation and geriatric psychiatry. HR personnel initiated the process by disseminating internal organisational announcements and screening their databases to identify eligible nurses. The research team had no prior connections with the participating nurses or the organisations they represented. Organisations and participants were informed that the study was undertaken for academic purposes. HR departments provided details of potential participants and the primary researcher contacted them. This communication, facilitated via phone and email, was aimed at arranging the interviews. Participants were informed that the primary researcher was an experienced female academic interested in nursing and employment‐related issues.

Semi‐structured interviews were conducted by the primary researcher between December 2019 and May 2021. Interviews took place at participant‐preferred locations, ranging from their homes and workplaces to cafes. At the outset of each interview, the research purpose, use of data and assurances of anonymity and confidentiality were thoroughly explained. All participants gave informed consent to participate in the interview and be tape‐recorded. Notes were taken during the interviews by the primary researcher to enhance interpretation.

The interview protocol was based on the existing literature on nursing professional identity (Ten Hoeve, Jansen, and Roodbol 2014; van der Cingel and Brouwer 2021). The interviews were structured first to allow participants to freely describe their workplace experiences as older adult care nurses, followed by questions about what nursing in older adult care meant to them. Examples of interview questions include ‘What does your workday as a nurse look like?’, ‘How do you view nurses in the older adult care context?’ and ‘What does being a nurse working in care for older adults mean to you?’. Two pilot interviews were conducted, which helped refine the process. A total of 50 nurses participated in this study and one potential participant decided not to participate due to time constraints. The interview duration ranged from 26 to 83 min, with an average length of 45 min. At the end of each interview, the interviewer summarised the discussion to seek participant confirmability and subsequently transcribed the interviews.

### 3.4 Data Analysis

The primary researcher applied an inductive content analysis to the full interview transcripts (Tong, Sainsbury, and Craig 2007), a common method in nursing research (Pursio, Kankkunen, and Kvist 2023). Manifest data analysis (Graneheim and Lundman 2004) was employed to assign codes to data fragments. In practice, this meant that codes reflected the actual words mentioned by participants. Latent data analysis was then utilised to construct categories and themes based on the codes (Graneheim and Lundman 2004). In practice, the latent analysis meant that themes and categories were named based on what the codes expressed, that is, the researcher's interpretation of the codes. The research team discussed the codes, categories and themes multiple times until a consensus was reached. Data analysis was iterative, implying that interview transcripts were reviewed several times. The software Atlas.TI was used to assist with organising, storing and coding the interview transcripts. Participating organisations were presented with the findings to obtain feedback.

### 3.5 Rigour and Trustworthiness

**FIGURE 1:**
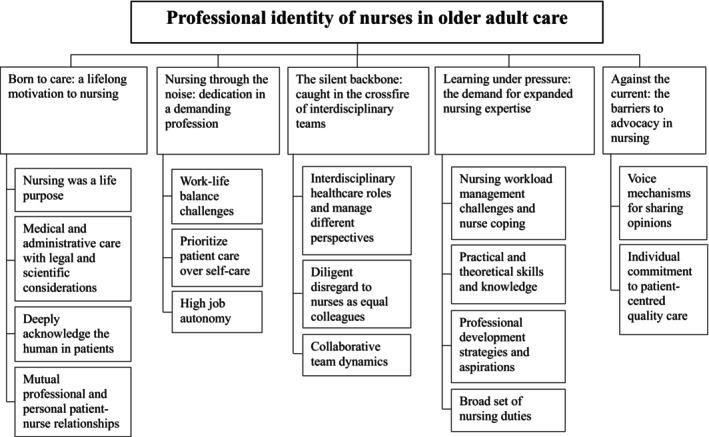
Themes and categories describing the nursing professional identity of nurses working in care for older adults.

Guidelines for ensuring rigour and trustworthiness in research were adhered to Graneheim and Lundman (2004). The team members conducted in‐depth discussions about the codes, categories, themes and distinctions to enhance the credibility of the research. To further enhance credibility, team members had diverse backgrounds, including PhD's in aged care, personnel management and professional nursing, leading to insightful discussions. To enhance transparency, participant quotes are presented in the findings to illustrate themes and a thematic framework is presented in Figure 1. Regarding participant credibility, participants had a wide variety of nursing experiences, which allowed them to provide a broad spectrum of perspectives on their professional identity.

The researchers, all female academics, aimed to enhance dependability by consistently conducting, transcribing and analysing each interview following a uniform protocol throughout the data collection and analysis process, continuing until data saturation was achieved. Data saturation was achieved when the interviewer observed that expressed experiences related to similar themes. The number of interviews enhances transferability by accommodating various lines of inquiry and reducing individual organisational bias.

### 3.6 Ethical Considerations

This study received ethical approval from Taylor's University Malaysia Human Ethics Committee in 2019 under approval code HEC 2019/052 when the lead author was affiliated with the institution. This study employed a procedure to access participants through healthcare organisations. Participating healthcare organisations were provided with full research details, including the study's objectives, data collection methods, data storage, the assurance of anonymity and confidentiality for both organisations and participants and the anticipated outcomes and advantages of participating in this study before giving their consent. Participating organisations delegated Human Resources (HR) personnel to assist in procuring participants. All participants were handed a detailed research information sheet which discussed the research objectives, assurance of confidentiality and anonymity, their rights and the voluntary nature of participation in the study.

## 4 Findings

### 4.1 Participant Characteristics

A total of 47 female and three male nurses participated in the interviews. Table 1 summarises participant demographic details, reflecting diversity in occupational tenure, with most employed for over 15 years (*n* = 28). Regarding age, most nurses were above 31 years old (*n* = 42). Most (*n* = 28) participants completed a Bachelor's degree in nursing and one completed a master's in Healthy Ageing. It is important to highlight that all participants were of Dutch descent. Regarding older adult nursing roles, most nurses were general nurses (*n* = 27) and area nurses (*n* = 12). General nurses supported and cared for older adults in larger nursing homes, providing inpatient care. Supervisory and coordinating nurses oversaw the organising healthcare plans for older adults and provided patient care. Quality assurance nurses checked whether older adults received enough care, assessed the type of care and whether care was delivered correctly. Area nurses provided extramural care and oversaw home healthcare, whereby they visited older adults with care needs who still lived independently.

**TABLE 1:** Demographic characteristics of participants.

Sex	Male: *n* = 3 Female: *n* = 47
Occupational tenure in years	0–5: *n* = 4 6–10: *n* = 8 11–15: *n* = 10 16–20: *n* = 9 21 and above: *n* = 19
Type of organisation employed	Older adults care: *n* = 50
Age in years	21–30: *n* = 8 31–40: *n* = 17 41–50: *n* = 12 51–60: *n* = 11 61 and above: *n* = 2
Highest completed degree	Vocational: *n* = 21 Bachelor: *n* = 28 Master: *n* = 1
Older adults nursing roles	General nurses: *n* = 27 Supervisory/coordinating nurse: *n* = 7 Quality assurance nurse: *n* = 4 Area nurse: *n* = 12

### 4.2 Professional Identity of Nurses Working in Care for Older Adults

Five themes were identified to reflect the professional identity of nurses working in care for older adults. A summary of selected quotes, codes and categories for each theme is provided in Table 2. Figure 1 summarises the themes and categories.

**TABLE 2:** A summary of a selection of quotes, codes and categories per theme.

Selected participant quotes	Selected codes	Selected categories	Themes
My family recognised the carer in me at my younger years, I care for family, friends, caring makes who I am. (P35)	A calling; the carer in me	Nursing was a life purpose	Born to care: a lifelong motivation to nursing
To care for people and prepare their healthcare plans. (P19) There are laws that prescribe nurses to raise the bedrail. (P27) Provide care based on scientific evidence. (P20)	Medical and administrative care; follow law; scientifically informed care	Medical and administrative care with legal and scientific considerations	
When it comes to patients, we do everything we can. (P45) We cannot let our patients down, we have to pull our weight together. (P23)	Ensure patients receive care, collectively overwork	Prioritise patient care over self‐care	Nursing through the noise: dedication in a demanding profession
Our personal mobile phone was constantly ringing. I was even afraid to pick it up, afraid they would ask me to go and work. (P15) They would call you in the weekend to discuss things. (P10) Still thinking about how to handle things at work when I sat on the couch in the evening. (P12)	Work continues at home physically and mentally, interrupts time off	Work–life balance challenges	
Above all, just do what the doctor says. (P6) When I called someone, for example, to get a blood sugar test done, it was always like a huge sigh like ‘we're busy, that's not possible now’. (P16)	Subordination to upper‐level decisions	Diligent disregard to nurses as equal colleagues	The silent backbone: caught in the crossfire of interdisciplinary teams
We can support each other, do things for each other, consult with each other, take each other's shifts when needed. (P33) At some point, you are doing the work for the team and not for yourself anymore. (P12)	Need others; team to fall back on; support each other; selflessness for the benefit of the team	Collaborative team dynamics	
More workload and more diseases of affluence in the department, but the number of nurses is not increasing. (P50)	Increasing workload for same number of nurses; burnout feelings	Workload management challenges	Learning under pressure: the demand for expanded nursing expertise
Trying to do the job well, backing up my arguments with theoretical knowledge. (P16)	Broad discipline knowledge, theory‐driven decisions	Practical and theoretical skills and knowledge	
We have e‐learning, upskilling, clinical classes and I involve myself in these for the benefit of the organisation. (P8) I want to do something that offers development prospects. (P4)	Continuous development; personal interest to develop; being human	Professional development strategies	
I'd like to say something when I feel I need to. (P10) I enjoy thinking about the quality of care, coming up with things that can be done better. (P6)	Care quality matters; personal drive to improve work aspects; have a say about something	Voice mechanisms for sharing opinions	Against the current: the barriers to advocacy in nursing
I was very irritated, how they treated us nurses, but more importantly how they treated the patients, that bothered me a lot, the quality of care was very poor. (P8) The quality of care is very important to me, patients need to receive good care from us. (P9)	Patients must receive quality care; individual drive to ensure quality	Individual commitment to patient‐centred quality care	

#### 4.2.1 Born to Care: A Lifelong Motivation to Nursing

This theme discusses nurses' innate, lifelong motivation to care for others, which is deeply rooted in the identity of the nurses described here. When participants were asked about the essence of their profession, their first responses were that they had always wanted to be a nurse and that this calling has been with them for as long as they can remember. They described nursing as something beyond a career choice, a vocation driven by the innate desire to care for others. Participant 14 said, ‘To care and do things for other people is something I desperately want to do’, while Participant 29 referred to nursing as ‘an extra dimension to my life’.

Participants also emphasised that ‘human involvement’ (P48) formed their professional identity, describing this as a desire to care for people as humans.

I view and treat older adults as human beings like us, not only performing simple nursing tasks but checking the full living circumstances of an older adult, considering their family and social, dietary and physical needs. (P34)

Participants further emphasised that care included providing physical tasks, such as administrating infusions, inserting central catheters and providing daily living activities, as well as administrative duties, such as maintaining patients' healthcare records and developing short and long‐term healthcare plans. A participant expressed, ‘We do so much more than just washing patients, such as feeding them, changing their clothes and managing ongoing healthcare plans!’ (P19). Numerous participants explained their broad range of duties, such as developing healthcare plans, discussing tasks and healthcare plans with colleagues and families, administering medication and having social conversations with older adults: ‘I am in charge of the planning of patient care, assessing patient care needs, and also providing patient care’ (P19), ‘Developing a social connection with patients is part of the care I provide’ (P20) and ‘Ensuring care processes in my nursing team are managed effectively’ (P22). The diverse care needs of older adults mean that nurses in care for older adults must possess both practical and theoretical knowledge.

Older adults have illnesses otherwise they would not be living with us. So, we must become familiar with the scientific knowledge about these illnesses to understand how to behave, treat and work with an older adult. My satisfaction with being a nurse comes from both helping an older adult live their life as comfortably as possible and, at the same time, broadening my scientific knowledge of older adult illnesses. (P26).

Participants revealed that they develop both professional and personal relationships with their patients. For some, the depth of these relationships became apparent when they cared for certain patients over extended periods, leading them to sometimes form personal attachments.

Patients become attached to us nurses as they share everything with us and I, as a nurse, also become attached to patients when my personality matches that of the patient. (P21)

Although not all participants intentionally build such deep relationships, they recognise them as inherent to working in care for older adults. Few participants expressed concerns about the closeness of these connections, ‘I knew them personally for an extended period, which made the relationship too personal, and this made me feel overwhelmed at times when older adults became emotional’ (P32). Other participants perceived these bonds as invaluable.

This patient found it difficult to be open to people about his emotions, but I noticed he showed his emotions to me, embraced me and it was like, wow, this is valuable. (P47)

Engaging on a personal level with patients was seen as a treasured experience by numerous participants. One participant expressed, ‘I believe it is important to develop a close connection with patients, and I work hard to achieve this’ (P48). Their dedication often extends beyond medical and clinical responsibilities. One participant emphasised her commitment to overall patient well‐being: ‘I'm not only putting a bandage, I also peek inside their fridge to check if there is enough food’ (P14). Another participant embraced social conversations with older patients and expressed:

A 10‐min small talk where I sit down, look at the patient and engage deeply in the conversation by listening to their concerns can sometimes prove more valuable to patients than a technical nursing treatment. (P36)

Other participants expressed a sense of fulfilment in caring, stating, ‘I enjoy providing all aspects of care, including “technical” nursing activities and using conversation techniques to make patients feel better’. (P46). Many participants agreed that they preferred to care for patients for a longer period due to having an interest in getting to know patients personally. Some nurses had previous nursing experiences in hospital settings where they felt that care for older adults was rushed with little opportunity to get to know a patient due to the quick turnover of patients.

In the hospital care setting where I used to work, I had to say goodbye to patients every week because they had finished their treatment, so I never really had a personal connection with patients, an aspect I deeply missed as my personal view on nursing care includes this social aspect as well. (P6)

Going beyond their prescribed call of duty underscores the depth with which nurses perceive and attend to older patients, viewing them as human beings with medical and social needs.

#### 4.2.2 Nursing Through the Noise: Dedication in a Demanding Profession

There was general agreement among all participants that nursing in care for older adults is demanding, affecting their work–life balance. The challenge that most participants identified was the unofficial duty to remain on standby 24/7, said to be primarily caused by national nurse shortages affecting the number of nurses available in their organisations.

There is a chronic national shortage of nurses and we are short of nurses at this older adult care facility, too. Our schedule is a huge challenge and keeps getting challenged by endured nurse shortages and long‐term absences from work, such as colleagues falling ill and pregnancies. This results in fewer nurses who are available and must fill all the gaps in our care schedule. (P44)

Many participants reported receiving unscheduled phone calls from colleagues who wanted to discuss patient updates outside official working hours, including weekends. While perceived as disruptive, this was considered an inherent aspect of their profession. Numerous participants reported their mobile phones ringing nonstop, leading to hesitancy in answering out of fear of being called back to duty.

I took my work home with me because I had a work phone, which allowed me to check updates and be on call. While I did not intend to use the phone at home, I often checked it as an unconscious automatic response to check how my patients were doing. In addition to this, they often called me asking if I could take on extra shifts. (P37)

Participants emphasised the great value they place on patient care, expressing, ‘We cannot let patients down’ (P25) and ‘We will do everything we can to ensure patients are cared for’ (P46) and candidly admitted that patient care often takes precedence over their self‐care. Participants shared that they often needed and wanted to take on additional shifts and night shifts to ensure the continuity of older adult care. At the same time, they knew working these extra shifts impacted their family members, particularly partners who had to manage their children's school drop‐off and bedtime arrangements. Some participants worried about the trend of nurses frequently overworking to meet older adults' care needs.

I noticed colleagues were overworking voluntarily, I'm not going to work extra hours if I don't get anything [money] back for it […]. We should not collectively voluntarily overwork, they don't do that in other occupations either. (P48)

Their prioritisation of patient care over self‐care is not solely due to their sense of duty; it also stems from the thoughtful autonomy and trust their profession bestows upon them. Participants stressed the value of personal and professional commitment to care for older adults, attributing it to their job autonomy. For these participants, autonomy meant freedom in care delivery choices, continually improving methods and implementing improvements in practice.

I enjoy being allowed to make many patient care decisions independently and value the autonomy the organisation gives us nurses so that we can deliver patient care up to the highest standards. (P16)

I completed a professional training and now apply what I've learned in my daily nursing practice. I also explain to nursing colleagues the social techniques I've learned from this training, which has improved the quality of many of our daily social patient–nurse interactions. (P18)

Some participants particularly valued their freedom to work independently, emphasising the flexibility to schedule their daily routes and the possibility ‘to use creativity to cope with an unforeseen situation’ (P19). The above experiences show that nursing in older adult care influences the well‐being of nurses given the demanding work schedules, while the autonomy nurses experience helps balance the intensity of their work.

#### 4.2.3 The Silent Backbone: Caught in the Crossfire of Interdisciplinary Teams

Participants expressed a dual nature of their position as nurses, one in which they experience the role of nurses as unrecognised, that is, the silent backbone and one in which they have to manage conflicting demands within interdisciplinary teams, that is, caught in the crossfire of interdisciplinary teams.

All participants viewed themselves as professionals in interdisciplinary teams and mentioned working with general practitioners and their assistants, municipality workers, voluntary workers, social workers, psychologists, physiotherapists, pharmacy workers, ergo therapists and doctors.

Our role as nurses in this older adult care facility is to reduce the care burden home visiting doctors have, so before the doctor's visit, we see the patient and perform nursing care such as placing IV pumps. (P28)

Several times a month, we discuss care processes and our ways of communicating with general practitioners. (P41)

While many participants find it rewarding to be part of interdisciplinary teams, some admitted feeling a sense of depersonalisation within the larger healthcare system, described as ‘feeling like a number’ (P30). Yet, in the face of systemic pressures, nurses are far from mere ‘cogs in the machine’. As part of their professional identity, they are not only responsible for overcoming frustrations, but also for providing unwavering attention to patient care. Nurses frequently find themselves at the crossroads of essential decision‐making and patient advocacy when providing care and when it comes to treatment choices. They ensure that compassion and expertise are combined in care delivery. Concerning patient advocacy, nurses expressed:

Speaking up for patient needs is very important to me as a nurse and person and I will not let the crowd influence my nursing values and beliefs or influence what I think is best for the patient. (P6)

Older adults who have had a stroke are often not able to say what they want to say at crucial moments, so I make sure I understand what they want before crucial conversations so I can speak on their behalf in such a conversation in an honest and professional manner. (P15)

Others felt caught in an intermediary position, managing varied expectations and requests from other healthcare teams who were not directly engaged in the day‐to‐day provision of older adult patient care. This indicates the multifaceted and interdisciplinary roles nurses in care for older adults undertake, requiring them to balance and manage a range of perspectives.

Seven different disciplines were located at different organisational levels and all wanted something from me and no one was on the same page. They pressured me to follow their suggestions, but even for simple things, there were so many different opinions, with one person saying ‘Do this’ and the other person saying ‘No, do that instead’ and I was caught in the middle. It felt like being kicked from different angles. (P47)

These frustrations do not define the entirety of nurse experiences. Nurses are frequently the ones who keep the team together, coordinating care and treatment across disciplines with a strong focus on patient outcomes. Experiences included, ‘It is a positive experience working in an interdisciplinary team; communication is smooth here, which has a positive impact on the overall care’ (P34) and ‘We wanted to improve the communication between us nurses and general practitioners, and recently we concluded that this is going much better due to our efforts improving collaboration’ (P10).

Participants also highlighted feeling overlooked and undervalued within interdisciplinary teams. Instead of being silenced by these challenges, nurses sharpened their advocacy skills by actively pushing for the best patient outcomes and, at times, involving themselves in interdisciplinary discussions. Nurses expressed: ‘I told my employer about working conditions that go against legal employment agreements’ (P15) and ‘Several colleagues have physical health concerns due to heavy patient lifting, so I decided to stand up for our rights and those of the patient and raised the need for proper lifting equipment so that we nurses can lift our patients safely’ (P37). While their advocacy may sometimes go unnoticed, it remains a powerful force that silently redefines healthcare boundaries.

A ‘hierarchical structure’ (P6) was reported, where participants found themselves following the orders of the management and doctors. When participants requested specific healthcare resources from other departments, some experienced ‘A sense of being perceived as annoying to the other departments’ (P16). This led to a pervasive feeling of being dismissively disregarded instead of being recognised as equal colleagues. The healthcare system does not solely impose this sense of undervaluation, it also arises from within the nursing profession and the individual nurses themselves (Ten Hoeve, Jansen, and Roodbol 2014). One participant expressed, ‘When I ran into things I didn't agree with, I often thought ‘never mind” (P29). This suggests that self‐imposed limitations also shape nurses' professional identity. Nurses are beginning to recognise these internal barriers and are working to dismantle them. By confronting these internal and external challenges, they are transforming moments of being overlooked into opportunities to reinforce and redefine their professional identity.

Participants underscored the importance of being part of a nursing team, which supported their decision‐making processes, which would have been challenging to undertake individually. In these teams, addressing ageism was a key aspect of their advocacy work, ensuring that older adults' rights and their dignity were always considered. This collaborative approach not only reinforced their advocacy efforts but also provided a dependable support system where team members could rely on each other at difficult times. Participants expressed their enthusiasm for assisting and receiving support from colleagues when necessary, expressing ‘It's amazing to receive support from nursing colleagues’ (P8) and ‘I help my nursing colleagues in completing assessments’ (P18).

Collectively, these experiences point to the presence of somewhat challenging collaborative team dynamics due to the presence of hierarchy and power dynamics stemming from healthcare professionals in ‘higher’ positions. Nurses valued collaborating with higher‐positioned healthcare professionals as it made them feel important, but they experienced little power when decisions were made.

#### 4.2.4 Learning Under Pressure: The Demand for Expanded Nursing Expertise

Participants expressed feeling overwhelmed by their daily workload, reporting that care became more complex due to increased diseases of affluence and challenges from working in an understaffed nursing department. Nurses narrated, ‘I am continuously updating my knowledge about older adult diseases and required treatments’ (P18), ‘We need more nurses as older adult care becomes more complex, so care processes take more time’ (P48) and ‘The huge nursing shortages drain the collective nursing workforce energy’ (P11). Another factor contributing to a demanding workload includes the ageing of the nursing workforce. Some older nurses are having difficulties to use new technologies and to work in a fast‐paced environment. In contrast, for younger nurses, this led to frustrations, for example, because they need to explain the use of technology to older nurses. Despite these frustrations, nurses stressed their ability to handle an excessive workload, both mentally and physically, described by one participant as the ‘need to have both feet steadily on the ground’ (P30). Challenges related to workload management were perceived as an inevitable aspect of the nursing profession, given that none of the contributing factors—like nurse shortages and an ageing population—are expected to be addressed in the foreseeable future.

To ensure complex care delivery, participants reported their imperative of continuous development in their practical and theoretical skills and knowledge. They expressed that improving these skills and knowledge enables them to think ahead of time and anticipate potential issues that prevent older adults from deteriorating further.

It is important to have a solid understanding of older adult illnesses and medications. We need to recognise and treat illnesses for which we must know what symptoms to focus on and why, justifying our decisions with theoretical knowledge. (P17)

Participants commended their employing organisations for offering many ways to maintain and broaden their knowledge and skills, describing, ‘There are lots of development opportunities here’ (P18). E‐learning, clinical classes and technical skills and knowledge evaluations were among the common professional development strategies offered. Besides these trainings, participants valued flexibility in training topics given the changing landscape of older adult care needs.

I am a person who enjoys learning and in light of the changing care landscape, I search for training that allows me to learn care aspects that go beyond the standard training packages the organisations offer to prepare myself for other unexpected future care needs. (P49)

Participants regarded themselves as performing diverse nursing roles. Beyond patient care, they were involved in many administrative and personnel management tasks, such as managing nursing schedules, sick leave conversations, annual staffing plans, job interviews and performance evaluations and preparing financial budgets.

I participate in recruitment and keep track of production figures, whether enough money is brought in and check to what budgets nurses have assigned care tasks. (P2)

We had a sick leave percentage of 18%, which was extreme compared to national sick leave percentages of around 4%. I was non‐stop busy trying to resolve gaps in nursing schedules. (P18)

These experiences highlight the need for nurses to upskill themselves and enhance their knowledge about older adult diseases and treatment plans to stay up‐to‐date in their nursing practice. In addition, nurses have become responsible for expanding administrative work, requiring diverse administrative and 21st Century (technological and digital) skills.

#### 4.2.5 Against the Current: The Barriers to Advocacy in Nursing

Participants expressed a desire to improve the workplace, voicing sentiments like ‘I enjoy sharing my ideas, and at my current employer, I get the freedom and trust to work out my ideas, particularly about improving care quality’ (P6) and ‘We get involved in opportunities to share our thoughts, allowing us to speak up’ (P17), indicative of their eagerness to voice their thoughts. Some participants appreciated brainstorming with colleagues to enhance work and care quality. They emphasised that they did not step back in raising their concerns, asserting, ‘The care quality given to patients is one of the aspects closest to our hearts as nurses’ (P15). These mechanisms for sharing perspectives are deemed one of the most important aspects of nursing advocacy. Some participants experienced resistance and underappreciation from higher leadership levels.

If you stick your head above the mowing field, your head will be cut off, it was not appreciated if you spoke up. (P14)

The patients in geriatrics and traumatology were all above 80 years old and were puzzled‐minded. At night, I used to walk in the corridors by myself to provide patient care and there would then usually be around three patients looking for the exit or bus stop. It was difficult to get them back to bed by myself and I found such situations dangerous for myself too. I complained to my manager about these situations, saying that they were irresponsible for patients and dangerous for us nurses; well, then I was seen and treated as the black sheep. (P39)

Being labelled as the ‘black sheep’ is only part of the story. Many nurses are relentless in their pursuit of excellence, turning setbacks into fuel for innovation. Though their advocacy may not always be on the front line, it is the steady force that drives healthcare forward.

I formed a group of nurses within our department who all thought alike about an issue we faced and we decided to email upper management about our concerns. (P14)

I am a driving force in helping fellow nurses to better understand and respond to patients who have dementia, which benefits patient well‐being and behaviour. (P31)

We are taking responsibility for improving departmental medication safety and helping fellow nurses to become skilled in medication safety. (P33)

The value participants assigned to quality care delivery was closely related to the presence of avenues for voicing their concerns. Some participants said they were not provided sufficient hours to deliver the care an older adult patient genuinely required. They felt rushed due to heavy patient loads and believed healthcare plans often fell short of actual care needs, breeding deeply felt personal frustration. For other nurses, such scenarios triggered an intrinsic motivation to teach older adults basic care activities. Nurses expressed they did so when they judged the patient to be capable of performing basic self‐care activities so that older adults could remain more independent and less reliant on care providers.

Frustration is, however, only one side of the coin. Despite everything, many nurses have shown incredible resilience and have used their extensive knowledge to fight tirelessly for their patients and the nursing profession as a whole and spoke about times when their advocacy led to real improvements in the quality of care.

This organisation is keen to innovate care processes based on our ideas. One day, I provided my ideas and the next day, the organisation was already implementing them; they wanted to be frontrunners in care innovation. (P22)

This shows that nurses keep creating spaces where their views are heard and their contributions are felt, even though the system does not always recognise them. These moments of success are no anomalies and demonstrate the critical role that proactive nursing advocacy plays in elevating care standards.

## 5 Discussion

This study investigated the professional identity of nurses caring for older adults. Nurses' perceptions of their professional identity shed light on the multifaceted nature of nursing in older adult care and their significant role in patient well‐being. Contrary to the perception of nursing as merely a simple and predominantly female job (van der Cingel and Brouwer 2021), the themes extracted from this study articulate a unique professional identity of nurses working in care for older adults. The professional identity of older adult care nurses portrayed in this study aligns with broader care philosophies, such as person‐centred care (Mayer et al. 2020; Phelan et al. 2020), based on four broad observations.

First, nurses working in care for older adults often go beyond their prescribed roles to care for older adult patients by recognising human needs in addition to medical needs, such as ensuring sufficient groceries and engaging in conversations. This behaviour is indicative of nurses' personal concern for patients. Second, despite participants feeling internal resistance to working during their off time, they remained approachable to discuss older adult patient updates, which reflects the value they place on caring for a dependent human being. Third, nurses working in care for older adults employ creativity and flexibility to improve how they provide care that best meets older patients' needs and desires, illustrating their ability to provide holistic care. Fourth, these nurses are concerned with enhancing their skills to ensure they are prepared for the increased demand and complexity of older adult patient care, demonstrating their drive to remain professionally competent. This commitment to comprehensive care also includes addressing ageism and ensuring that older adults are treated with respect and dignity.

The person‐centred care philosophy (Byrne, Baldwin, and Harvey 2020; Mayer et al. 2020; Phelan et al. 2020) emphasises the importance of putting the people aspect of patients first and acknowledging the professional as a person in the care relationship. Having explored professional identity in the context of care for older adults, our study adds nuance to person‐centred care philosophies by considering the degree of power nurses have in the care relationship with older adults. It can be challenging for nurses to fully accommodate older adults' preferences for care delivery due to gerontological conditions such as dementia and delirium. Therefore, nurses in older adult care often take a more active role in directing patient care while practicing creativity to meet older adults' care needs. Additionally, the social relationship between patients and nurses in older adult care often extends into nurses' personal lives due to the significant length of time and dependency of older patients on their assigned nurse.

The broad spectrum of aspects forming nurses' professional identity suggests that nursing in older adult care deserves recognition as a professional occupation. Part of how the public portrays nurses in care for older adults is influenced by how nurses in these settings portray themselves. In this research, most nurses first emphasised ‘caring for others’ as core to their professional identity. While nurses in our study interpret ‘caring for others’ in numerous ways, the public perceives ‘care’ as a simple and compassionate task (van der Cingel and Brouwer 2021). This misalignment between nurses' interpretation of ‘care’ and the public's view implies a pressing need for nurses working in older adult care to reshape public perceptions, for example, by emphasising their comprehensive technical and practical skillsets, possibly through social media outreach.

While the views of nurses on professional identity in this study align with those of other types of nurses (Brzozowski, King, and Steege 2023; Dahl and Clancy 2015; van der Cingel, Andela, and Barf 2023), they distinctively describe caring for older adults as a personalised form of nursing. This personalised form of nursing was sometimes problematic, as nurses became too heavily involved personally in patient care, leading to work–life balance challenges. Such personal involvement was sometimes discussed as leading to involuntary overwork, which could result in burnout. Training in setting boundaries is essential for nurses working in older adult care to prevent burnout. Jefferies et al. (2023) discovered that nurses who engage in self‐care strategies, such as exercising and maintaining a social network outside of work, can transition more smoothly from work to leisure. As a result, they are not preoccupied with work during their time off, leading to a greater sense of being well‐rested before returning to work.

Similar to primary care registered nurses (Brzozowski, King, and Steege 2023, 11), nurses in this study noted the evolving nature of their profession, particularly the increased care complexity, which necessitates ongoing learning. This underscored nurses' intrinsic motivation to update and refine their skills and knowledge. Offering nurses a stimulating and psychologically safe learning environment, allowing time for reflective questioning and case discussions among nurses, is crucial (van der Cingel, Reinders‐Messelink, and Stallinga 2023). It is also important to acknowledge that increased care complexity and the subsequent need for nurses to enhance their skills create a vicious cycle where nurses are continually prompted to participate in training, increasing their workload. Given the demanding nature of nurses workloads (Clayton and Marczak 2023), continuous training participation adds a burden that could adversely impact nurses well‐being and quality of life (Babamohamadi et al. 2023; Xiao, Cooke, and Chen 2022). Nurse leaders should consider nurses' involvement in such training when allocating workload.

Another issue that surfaced in this research was the lack of recognition that nurses working in care for older adults receive from fellow healthcare professionals. While Pursio, Kankkunen, and Kvist (2023) recommend that nurses be part of multi‐professional groups at an organisational level to improve their professional identity, our findings suggest that nurses often feel undervalued in these settings due to how other healthcare professionals interact with them. Nurses are trained to aim for high‐quality care in their nursing qualifications, but when they advocate for this to other healthcare professionals, their efforts often remain unacknowledged. This finding aligns with research suggesting that healthcare organisational hierarchy affects communication effectiveness between nurses and other hierarchically higher‐positioned healthcare professionals (Aspinall, Jacobs, and Frey 2021). This is problematic because a lack of recognition from higher‐positioned healthcare professionals disempowers nurses. George et al. (2023) found that hospital nurse empowerment is enhanced when they are invited to speak up about their concerns and receive appreciation from nursing leaders and upper management. Societal gender norms also seem to influence nurses' feelings of being undervalued (George et al. 2023). To date, nursing remains a female‐dominated profession and is subject to societal pressures to conform to gender norms. This affects the degree of power female nurses perceive, for example, in influencing change in a context dominated by male leadership (Punshon et al. 2019). Sector‐wide policy reforms outlining the interdisciplinary role of nurses are needed to ease inter‐level communication and stimulate collaboration. Initiatives for promoting diversity and inclusion within the nursing profession are also needed, such as public awareness campaigns that challenge gender stereotypes in nursing.

This study lays bare a pressing issue: the profound disconnect between the multifaceted roles of nurses caring for older adults and the lack of public recognition, including recognition from fellow healthcare professionals. Given the challenges of an ageing population, nurse shortages and high turnover, there is no room for this misalignment. Policy reforms paired with a societal shift in recognising nursing in older adult care as a professional occupation are crucial to ensuring the profession receives the recognition and value it rightfully deserves.

### 5.1 Strengths, Limitations and Suggestions for Further Research

Formulating nurse identity from 50 interviews with nurses working in care for older adults is seen as one of the main strengths of this study. The dataset were sufficiently broad to capture many aspects of nurses' professional identity. With every study, there are limitations to be noted. Firstly, this study only includes the perspectives of nurses who worked in care for older adults, so the findings do not necessarily reflect how nurses in other healthcare settings, such as primary care, hospitals and ambulatory care, view their professional identity. Understanding variations across nursing is important and has the potential to enhance nursing education, enabling student nurses to understand better and align themselves with the nuances of different nursing domains. One limitation to consider is that the dataset is skewed towards female participants. While no discernable differences in responses were noted between male and female participants, a more balanced representation of both genders could have unveiled variations in identity nuances across genders. Future research could consider employing a differentiated sampling strategy, specifically targeting equal numbers of male and female nurses, to balance the gender dynamics in the sample. HR could use insights from such a study to develop refined recruitment strategies to attract more males and females to the profession.

This study was also limited to Dutch nurses working in healthcare organisations based in the northern part of the Netherlands—this region has less ethnic diversity than other parts of the Netherlands, such as the West. Hence, the study sample is not ethnically diverse. Since the Netherlands is considered a developed country, our findings do not reflect nurse identity from a developing country nursing context. Many nurses from developing countries seek employment in developed countries, with countries initiating nurse recruitment programmes targeting migrant nurses to work in older adult care. Understanding migrant nurses' perspectives on older adult care will enrich the discourse on the nursing professional identity. Also, the study did not assess the role of participant personality in how participants viewed themselves in the nursing profession, so further research is needed to evaluate the extent to which nurses are responsible for creating the public perception about nursing. Lastly, this research was situated at a single point in time and employed a single data collection method, which could have resulted in bias. Future studies could implement triangulation methods to mitigate this bias, such as using multiple data sources and collecting data at multiple intervals to validate and enrich the findings.

## 6 Conclusion

Healthcare organisations are facing significant nurse shortages which pose challenges to the functioning of healthcare systems. With ageing populations and increased complexity of care, there is heightened pressure on older adult care. This situation necessitates the employment of more nurses working in older adult care equipped with the skills needed to meet future care demands and complexities. Understanding the nursing professional identity can be a powerful motivator for individuals to enter and remain committed to the profession. This study's findings elucidate the meanings nurses working in older adult care assign to their professional identity. It is imperative for both healthcare organisations and nurses to elevate public perceptions of nursing in care for older adults and to wholeheartedly embrace the professional nursing identity highlighted in this study. Older adult care organisations and nurses should actively engage in public outreach initiatives to share their professional experiences and debunk antiquated stereotypes.

## Author Contributions

All authors made substantial contributions to the conception and design, acquisition of data, or analysis and interpretation of data; Involved in drafting the manuscript or revising it critically for important intellectual content; Given final approval of the version to be published. Each author should have participated sufficiently in the work to take public responsibility for appropriate portions of the content; Agreed to be accountable for all aspects of the work in ensuring that questions related to the accuracy or integrity of any part of the work are appropriately investigated and resolved.

## Conflicts of Interest

The authors declare no conflicts of interest.

### Peer Review

The peer review history for this article is available at https://www.webofscience.com/api/gateway/wos/peer‐review/10.1111/jan.16506.

## Supporting information


**Appendix S1.** Supporting Information.

## Data Availability

The data used in this study is confidential.
